# PReS-FINAL-2023: Uveitis surveillance through lean-six sigma for quality assurance in juvenile idiopathic arthritis

**DOI:** 10.1186/1546-0096-11-S2-P36

**Published:** 2013-12-05

**Authors:** A Patwardhan, K Kelleher, K Jones, S Ardoin, C Spencer

**Affiliations:** 1 Pediatric Rheumatology, Ohio State University, Columbus OH, USA; 2 Pediatric Rheumatology, University of Misouri School of Medicine, Columbia, MO, USA; 3 Research division, Ohio State University, Columbus, OH, USA; 4 Pediatric Rheumatology, Nationwide Childrens Hospital, Ohio State University, Columbus, OH, USA

## Introduction

Chronic asymptomatic iridocyclitis occurs in 10-20% of all patients with JIA leading to insidious but progressive morbidity and possible blindness. Patients with JIA-associated uveitis need to be seen by an ophthalmologist regularly. Lean thinking is based upon the following principles 1) Identifying the customer value (value adding steps) and alleviating the redundant parts of the process hence minimize waste 2) developing an effective flow production 3) eliminate backflows 4)using "pull" techniques 5) striving to perfection.

## Objectives

To use Lean methodology to develop uveitis surveillance process for JRA patients for the electronic health records.

## Methods

Various tools of lean methodology were used throughout the development of a new process of uveitis surveillance for JIA patients visiting rheumatology clinics at Nationwide Children's Hospital. Problems were identified after paper chart review of 400 JIA patients. The key performance indicators used were 1) number of patients given eye examination request sheet 2) number of eye examination results received back from eye doctors & 3) number of eye examination results documented and available during the clinic visit. The hospital has switched to electronic medical records (EPIC) since 2006. It was found on baseline data that uveitis surveillance was inadequate and ineffective in paper charts. We identified the need to develop an electronic health record - based new surveillance process which can be more effective in improving communication between eye doctors, rheumatologists and patients/parents. We performed value stream mapping by stake holders to sketch the initial process and identify bottlenecks. Delphi survey was then used to reach consensus decisions, though out the project time. We charted current state, future state & ideal state and performed gap analysis. We then developed a pareto-matrix. The project methodology was based on the Deming's PDSA cycles. We developed a standard work based on our initial PDSA cycles. We evaluated the new process through kpis.

## Results

The uveitis surveillance process improved inter-team communication and quality of care. Inbuilt alerts in the process for presence of eye disease and missed eye appointments prompted rheumatologist to take appropriate timely action.

## Conclusion

Implementation of lean tools and thinking can make provide smarter, quicker, easier, better and safer uveitis patient care delivery to the JIA patients by use of an effective uveitis surveillance process. We also emphasize the importance of seeing lean thinking as a part of the larger management shift towards planning for changes in mindsets and work places. This new surveillance process can be horizontally deployed for diabetic eye surveillance and drug toxicity monitoring in rheumatic patients on immunosuppressant.

## Disclosure of interest

None declared.

